# Do children report differently from their parents and from observed data? Cross-sectional data on fruit, water, sugar-sweetened beverages and break-time foods

**DOI:** 10.1186/s12889-016-2963-7

**Published:** 2016-04-18

**Authors:** V. M. van de Gaar, W. Jansen, M. J. J. van der Kleij, H. Raat

**Affiliations:** Department of Public Health, Erasmus University Medical Centre, P.O. Box 2040, 3000 CA Rotterdam, The Netherlands; Department of Social Development, City of Rotterdam, P.O. Box 1024, 3000 BA Rotterdam, The Netherlands; Department of Public Health & Primary Care, Leiden University Medical Centre, P.O. Box 9600, 2300 RC Leiden, The Netherlands

**Keywords:** Children, Parents, Observed behaviour, Sugar-sweetened beverages, Fruit, Water, Snacks, Break-time foods, Agreement

## Abstract

**Background:**

Reliable assessment of children’s dietary behaviour is needed for research purposes. The aim of this study was (1) to investigate the level of agreement between observed and child-reported break-time food items; and (2) to investigate the level of agreement between children’s reports and those of their parents regarding children’s overall consumption of fruit, water and sugar-sweetened beverages (SSB).

**Methods:**

The children in this study were 9–13 years old, attending primary schools in Rotterdam, the Netherlands. Children were observed with respect to foods brought for break-time at school. At the same day, children completed a questionnaire in which they were asked to recall the food(s) they brought to school to consume during break-time. Only paired data (observed and child-reported) were included in the analyses (*n* = 407 pairs). To determine each child’s daily consumption and average amounts of fruit, water and SSB consumed, children and their parents completed parallel questionnaires. Only paired data (parent-reported and child-reported) were included in the analyses (*n* = 275 pairs). The main statistical measures were level of agreement between break-time foods, fruit, water and SSB; and Intra-class Correlation Coefficients (ICC).

**Results:**

More children reported bringing sandwiches and snacks for break-time than was observed (73 % vs 51 % observed and 84 % vs 33 % observed). The overall agreement between observed and child-reported break-time foods was poor to fair, with ICC range 0.16–0.39 (*p* < 0.05). Children reported higher average amounts of SSB consumed than did their parents (1.3 vs 0.9 L SSB, *p* < 0.001). Child and parent estimations of the child’s water and fruit consumption were similar. ICC between parent and child reports was poor to good (range 0.22–0.62, *p* < 0.05).

**Conclusion:**

Children report higher on amount of break-time foods as compared to observations and children’s reports of SSB consumption are higher than those of their parents. Since the level of agreement between the observed break-time foods and that reported by children and the agreement of child’s intake between parent and child reports are relatively weak, future studies should focus on improving methods of evaluating children’s consumption behaviour or on ways on how to best use and interpret multiple-source dietary intake data.

**Trial registration:**

Current Controlled Trials NTR3400.

**Electronic supplementary material:**

The online version of this article (doi:10.1186/s12889-016-2963-7) contains supplementary material, which is available to authorized users.

## Background

Insight into children’s consumption habits is important for two main reasons. Firstly, it is widely known that eating and drinking habits can contribute to the development of childhood obesity [1]. Secondly, the consumption habits that we have as children continue into adulthood, when the risk of obesity remains [2]. Therefore, gaining insight into a child’s consumption habit is important. However, for assessing dietary behaviour on the level of pre-specified foods and food groups there seems to be no perfect measurement method [3].

Interventions aimed at changing children’s consumption behaviour are commonly evaluated using information on the habitual consumption behaviour of the child reported by parents [4, 5]. Unfortunately, research has shown that parents may not always be a reliable source of information on the child’s habitual intake of foods and drinks [6, 7]. In addition, social desirability, especially among parents, may lead to over-reporting of the intake of healthy food items and under-reporting of the intake of unhealthy food items [3].

The cognitive ability of children to self-report their intake of foods and food groups is also doubtful [8, 9]. The ability to self-report improves when a child grows older [5], with some suggesting that children should at least be ≥12 years old to report more reliably on their dietary intake [10]. Other research has shown that children from the age of 8 years old may already be reliable sources of information regarding their own food intake over the past 24 h [5, 8, 11, 12]. However, due to their unfamiliarity with concepts such as ‘frequency’ and ‘averaging’, it is debatable whether children of this age respond accurately to food frequency questionnaires when items cover longer periods [5, 8, 11]. Indeed, when Börnhorst and colleagues investigated the nature and magnitude of selective misreporting of food intake of different food items, they found children’s level of under-reporting to be 8.0 %, with over-reporting at 3.4 % [3]. Other studies have shown under-reporting of intake to be significantly higher in obese children [8, 11, 13]. These same studies have also demonstrated that not only does the extent of misreporting increase with age, in contrast to previous mentioned studies [5, 10]. Also, those who under-report on one occasion are likely to under-report on a second occasion, in which case reporter bias cannot be eliminated by repeated measures. Then again, research by Burrows and colleagues suggests that children between the ages 8 and 11 years old may report reliably as compared to either their father or their mother with regard to the child’s dietary intake of specific foods [14].

As can be concluded from the arguments above, all of the available methods for measuring dietary behaviour on the level of pre-specified foods and food groups may have some degree of misreporting and error [8, 11, 15–17], which makes measuring a child’s habitual consumption a challenging aspect of behaviour research. As previous research has indicated, in order for us to choose the most accurate measurement method for assessing children’s eating behaviour, we must improve our understanding of discrepancies between observed and reported behaviour [18–20].

The aim of this study was twofold: (1) to investigate the level of agreement between observed and child-reported break-time food items; and (2) to investigate the level of agreement between children’s reports and those of their parents regarding the children’s overall consumption of fruit, water and sugar-sweetened beverages (SSB).

## Methods

Our cross-sectional study used data from the population-based ‘Water campaign’ intervention [21]. This controlled trial aims to assess the effects of a combined school- and community-based intervention on children’s SSB consumption. The Medical and Ethical Review Committee of the Erasmus Medical Centre issued a ‘declaration of no objection’ (i.e. formal waver) for this study (reference number MEC-2011-183). Four primary schools located in multi-ethnic, socially deprived neighbourhoods in Rotterdam, the Netherlands, were included in the 'Water campaign' intervention study. This resulted in a total of 1288 children aged 6–13 years (grades 3–8) who were invited to participate. Passive parental consent was obtained. Parents (and children) received an information brochure to notify and inform them about the intervention and study participation. The study was also announced by the school, via the school letter and through the teachers and by flyers which were visible throughout the school. Parents (and children) were free to refuse participation without giving any explanation. They could do so by informing one of the teachers at their school or one of the researchers when present at school. At all times, the researchers could be contacted by a special phone-number or e-mail, for instance to decline participation [21].

The questionnaire items were based on previously widely used questionnaires, mainly used in earlier Dutch studies [22, 23]. We used two questionnaires to assess habitual consumption: one version was completed by children in grades 6–8 and another was completed by the parents of children in grades 3–8. Children filled in their questionnaire at school in the presence of a researcher and their teacher. The parent questionnaire was to be completed at home by the main caregiver of the child, within a period of maximum two months.

To objectively record what children brought to school to consume during break-time we used observation forms. These forms have been frequently applied for these kinds of information gathering by the Youth Health Care in recent years. Observations (unobtrusive) at school for children in grades 3–8 were conducted by trained public health professionals.

For the present study, we used baseline data from children in grades 6–8 only (aged 9–13 years). Pairing of data from the child questionnaires with data from the parent questionnaires or with data from the observations generated a study population of 539 children.

### Population characteristics

Socio-demographic characteristics were obtained from parent and/or child reports: the parent and child questionnaires included items on child’s gender, age, grade and ethnic background. Ethnic background was determined by country of birth of the parents according to definitions given by Statistics Netherlands [24]. The child’s ethnic background was defined as Dutch only if both parents had been born in the Netherlands; if one of the parents had been born in another country, ethnic background was defined according to that country; and if both parents had been born in different foreign countries, ethnic background was defined as the mother’s country of birth. Ethnic background was categorised as either Dutch, Surinamese/Antillean, Moroccan/Turkish or other/missing.

Gender, age and educational level of the caregiver were recorded. The caregiver’s highest educational level was categorised as either ‘high’; ‘mid-high’; ‘mid-low’; or ‘low’, based on standard Dutch cut-off points [25].

Trained personnel measured the child’s height and weight at baseline. Weight status was determined by calculating Body Mass Index in kg/m^2^ with height measured to the nearest 0.1 cm and weight measured to the nearest 0.2 kg, in light clothing or gym clothes, according to a national standardized protocol for Youth Health Care [26]. Children were categorised as being either ‘non-overweight’ or ‘overweight or obese’, based on cut-off points published by the International Obesity Task Force [27].

### Data pairs from observations and child reports

Trained public health professionals observed and registered which food items children brought along for the 10:00 am break at school. During the same morning, children completed a questionnaire in which they were asked to recall the food(s) they brought to school to consume during break-time. In Additional file 1: S1 and S2, the observation form and the child’s questionnaire item are shown. Data from the observations and child reports were grouped into three categories (‘sandwiches’, ‘fruit’ and ‘snacks’) by two researchers independently of each other. Any inconsistencies were discussed and where necessary a third researcher was included in order to reach agreement. If food items did not fit into one of the categories, these items were coded as ‘missing’ (<5 %). A ‘nothing’ category was added for those children who had brought nothing to eat during break-time. The four categories were dichotomised into (0) ‘not brought along’, and (1) ‘yes, brought along’. Paired data (observed and child-reported) were included in the analyses (*n* = 407 pairs).

### Data pairs from parent and child reports

Children and their parents completed parallel questionnaires regarding the child’s fruit, water and SSB intake. We assessed ‘daily consumption’ and ‘average amounts consumed’. Data collection took place over a period of two months, in April and May. Paired data (parent-reported and child-reported) were included in the analyses (*n* = 275 pairs).

Daily consumption of fruit was measured using the question 'Does your child/do you consume fruit on a daily basis?', with answer categories ‘no, not every day’ or ‘yes, every day’. Average amounts of fruit consumed was measured using the question ‘On a day your child eats/you eat fruit, how many pieces of fruit does your child/do you consume on average?’. Answer possibilities ranged from ‘half a piece of fruit’ to ‘two or more pieces of fruit’. Examples were given to assist respondents in determining the number of fruit pieces (e.g. tangerine as a half piece; an apple as one piece).

Daily consumption of water was assessed using the question ‘Does your child/do you drink water on a daily basis?’, with answer categories ‘no, not every day’ or ‘yes, every day’. Average amounts of water consumed was measured using the question ‘On a day your child drinks/you drink water, how many glasses of water does your child/do you consume on average?’. Answer possibilities ranged from ‘none’ to ‘five or more glasses of water’. The total water intake per day, converted to litres (for comparison with SSB), was calculated by multiplying the number of glasses by an estimated average volume of 200 ml.

Daily consumption of SSB was measured using the question ‘Does your child/do you drink SSB on a daily basis?’, with answer categories ‘no, not every day’ or ‘yes, every day’. Average amounts of SSB consumed was measured using the question ‘On a day your child drinks/you drink SSB, how many glasses (250 ml), cans (330 ml) or bottles (500 ml) does your child/do you consume on average a day?’. Answer categories ranged from ‘none’ to ‘five or more’. The total SSB intake per day, converted to litres, was calculated by adding up the volumes of the total number of glasses, cans and bottles that were consumed. Examples of SSB were provided, based on our definition of SSB: *Beverages containing added sugar, sweetened dairy products (*e.g. *chocolate milk), fruit juice (*e.g. *apple juice), soft drinks (*e.g. *cola) and energy drinks (*e.g. *sport energy drinks).* In Additional file 1: S3, an overview of the questionnaire items used to assess SSB intake are given.

### Analysis

For the dichotomous variables in the observed-child data pairs and parent-child data pairs, we calculated overall level of agreement (% same quartile). Kappa was used as an effect-size measure for the level of agreement, ranging from ‘0’ (agreement as expected by chance) to ‘1’ (perfect agreement) [28]. Agreement strength was based on the following criteria: 0.00–0.20 = ‘poor’; 0.21–0.40 = ‘fair’; 0.41–0.60 = ‘moderate’; 0.61–0.80 = ‘good’; 0.81–1.00 = ‘very good’ [29].

To explore the relationship between the different measurements methods with regard to consumption, Intra-class Correlation Coefficients (ICC) were calculated for each of the analysed behaviours. This generated a measure of absolute agreement for the dichotomous variables. For the continuous variables in the parent-child data pairs, the calculated ICC was a measure of consistency. The ICC is a value that ranges between '0' and '1', with a higher ICC corresponding to a better correlation. The following ICC cut-points were used: 0.00–0.20 = ‘poor’; 0.21–0.40 = ‘fair’; 0.41–0.60 = ‘moderate’; 0.61–0.80 = ‘good’; 0.81–1.00 = ‘very good’ [30, 31]. The mean (SD) of the difference and the limits of agreement were also calculated using Paired T-tests and used for input for the Bland Altman plots [32].

The McNemar test was used to compare the child’s reports with that of the observed reports or the reports by parents (level of significance set at 5 %) [32].

## Results

Table 1 provides information about several population characteristics. In the observed-child data pairs (*n* = 407), the children’s mean age was 10.6 years (SD 1.1), 49.5 % were girls, 22.9 % were of Dutch origin, and 22.6 % were overweight or obese.

**Table 1:** Characteristics of children and caregivers included in study

	Observed-child data pairs *n* = 407		Parent-child data pairs *n* = 275	
Child characteristics	*n*	Mean (SD) or %	*n*	Mean (SD) or %
Gender, % girls	200	49.5 %	150	54.5 %
*missing*	*3*	*0.7 %*	*0*	*0.0 %*
Age	406	10.64 (1.1)	274	11.06 (1.0)
*missing*	*1*	*0.2 %*	*1*	*0.4 %*
Grade				
- Grade 6	161	39.6 %	97	35.3 %
- Grade 7	78	19.2 %	101	36.7 %
- Grade 8	168	41.2 %	77	28.0 %
*missing*	*0*	*0.0 %*	*0*	*0.0 %*
Ethnicity				
- Dutch	93	22.9 %	56	20.4 %
- Surinamese/Antillean	97	23.8 %	56	20.4 %
- Moroccan/Turkish	118	29.0 %	90	32.7 %
- Other/missing	99	24.3 %	73	26.5 %
Weight status, % overweight/obese	89	22.6 %	68	25.9 %
*missing*	*13*	*3.2 %*	*12*	*4.4 %*
Caregiver characteristics				
Age	-	-	273	38.42 (9.1)
*missing*			*2*	*0.7 %*
Gender, % female	-	-	223	88.8 %
*missing*			*24*	*8.7 %*
Level of Education	-	-		
- High			45	16.7 %
- Mid-high			77	28.6 %
- Mid-low			74	27.5 %
- Low			73	27.1 %
*missing*			*6*	*2.2 %*

In the parent-child data pairs (*n* = 275), the children’s mean age was 11.1 years (SD 1.0), 54.5 % were girls, 20.2 % were of Dutch origin, and 26.0 % were overweight or obese. With regard to the caregivers, 88.8 % were female, their mean age was 38.4 years (SD 9.1), and 27.1 % were classified as having a low level of education.

### Observed-child data pairs

Table 2 shows the results of the analyses of the observed and child reports. Relative to observed reports of brought foods, the children themselves reported a higher amount of sandwiches (73.0 % vs 50.6 % observed) and snacks (83.8 % vs 33.2 % observed).

**Table 2:** Agreement between observed and child reports on food items that children brought to school with the intention to consume during break-time at school

Primary outcomes - *categories*	*N*	Number of times observed (%)^5^	Number of times reported by child (%)^5^	Overall agreement	Kappa^1,2^	*P*-value^3^	ICC^1,4^
‘Nothing’ (*brought nothing with them)*	407	16 (3.9 %)	7 (1.7 %)	95.8 %	.24 ***	0.049	.39 ***
Sandwiches	407	206 (50.6 %)	297 (73.0 %)	54.6 %	.09	<0.001	.16 *
Fruit & vegetables	407	76 (18.7 %)	39 (9.6 %)	78.1 %	.11 *	<0.001	.21 **
Snacks	407	135 (33.2 %)	341 (83.8 %)	38.1 %	.01	<0.001	.02

^1^Significance (2-tailed): *0.05 level, **0.01 level, ***≤0.001 level

^2^Cohen’s Kappa - corrected for agreement based on chance

^3^McNemar test

^4^Average Intra-class Correlation Coefficients (ICC) resembles measure of absolute agreement

^5^In case multiple food items were brought for break-time, the sum of the category percentages may exceed 100 %

The level of agreement was poor (total ĸ range 0.11–0.24, *p* < 0.05). The ICC between the observed and child-reported brought break-time foods was poor to fair (total ICC range 0.16–0.39, *p* < 0.05).

### Parent-child data pairs

Table 3 shows the mean and (SD) of average amounts of fruit, water and SSB consumed and the proportion who consume these items daily as reported by parents and children. In Additional file 1: S4, the Bland Altman plots are given.

**Table 3:** Agreement between parent and child reports on consumption of fruit, water and sugar-sweetened beverages (SSB)

Primary outcomes	*N*	Parent reported Mean (SD) or n (%)	Child reported Mean (SD) or n (%)	Difference Mean (SD)^3^	Overall agreement	Kappa^1,2^	*P*-value^3^	ICC^1,4^
Average amount of fruit consumed (# pieces)	252	1.42 (0.5)	1.48 (0.5)	0.06 (0.6)	-	-	0.150	.39 ***
Daily fruit % yes	250	117 (46.8 %)	99 (39.6 %)	-	56.8 %	.12 *	0.101	.22 *
Average amount of water consumed (L)	253	0.63 (0.3)	0.66 (0.3)	0.04 (0.3)	-	-	0.065	.59 ***
Daily water % yes	258	174 (67.4 %)	194 (75.2 %)	-	76.7 %	.44 ***	0.013	.61 ***
Average amount of SSB consumed (L)	253	0.92 (0.6)	1.33 (0.8)	0.41 (0.9)***	-	-	<0.001	.44 ***
Daily SSB % yes	258	141 (54.7 %)	83 (32.2 %)	-	58.1 %	.19 **	<0.001	.32 ***

^1^Significance (2-tailed): *0.05 level, **0.01 level, ***≤0.001 level

^2^Cohen’s Kappa - corrected for agreement based on chance

^3^McNemar test (dichotomous variables) or Paired *T*-test (continues variables)

^4^Average Intra-class Correlation Coefficients (ICC) resembles measure of absolute agreement (dichotomous variables) or consistency (continues variables)

Average amounts of fruit consumed reported by parents and children was similar in both groups and children reported a slightly lower daily consumption of fruit compared to their parents report. The level of agreement was poor (ĸ daily fruit = 0.12, *p* = 0.047), with overall agreement between children and parents of 56.8 %.

The average amounts of water consumed in litres reported by parents and children was similar. Children reported consuming water on a daily basis significantly more often as compared to their parents report. The level of agreement was moderate (ĸ daily water = 0.44, *p* < 0.001).

The average amounts of SSB consumed in litres reported by children was significantly higher than the volume reported by their parents. Children indicated consuming SSB on a daily basis also significantly less frequent than did their parents. Level of agreement was poor (ĸ daily SSB = 0.19, *p* = 0.001).

The ICCs between the child and parent reports were poor to good (total ICC range 0.22–0.62). For fruit consumption, the ICC ranged from 0.22 to 0.39 (fair ICC, *p* < 0.05) and for SSB consumption from 0.32 to 0.44 (fair to moderate ICC, *p* < 0.001); the ICC for water consumption was the highest, with a range of 0.59–0.62 (moderate to good ICC, *p* < 0.001).

The results are reported in accordance with STROBE (Strengthening the Reporting of Observational Studies in Epidemiology). See Additional file 2 for the STROBE checklist [33].

## Discussion

The level of agreement between the observed break-time foods and the children’s self-report was poor to fair: children reported higher quantities on their break-time foods of sandwiches and snacks. There was a poor level of agreement between the reports from parents and children regarding whether children consumed fruit and SSB daily, and a moderate level of agreement for daily water consumption. Despite these differences, on a group level children and parents did report similar average amounts regarding what the child consumes on a day and whether or not he/she drinks water or eats fruit, with the highest ICC for water (good agreement). The reports on average amounts of SSB consumed differed substantially between children and parents however.

Our result of 67 % overall agreement between observed and child-reported break-time foods is similar to that seen in previous studies. For example, in a study by Weber and colleagues, children were able to correctly recall 75 % of the school meal foods that observers had documented that they had on their plate [34]. Subsequently, Baranowski and colleagues found 83 % agreement between observed and recalled 12-h food consumption among children [35]. Despite this agreement, we also found differences, for example between observed and child-reported sandwich intake. The higher reported amounts by children could be explained by the fact that while observers only counted the sandwiches that children brought to school to eat during break-time at 10:00 am, some children may well have reported the number of sandwiches brought to consume during both break-time and lunch. Apart from this, our overall findings indicate that children also report higher amounts of their break-time snacks. A further possible explanation for this lack of agreement is that children find it difficult to estimate the amount of foods and food items, as other studies have reported [8, 9, 11].

Whereas on a group level the reports from parents and children regarding average amounts of fruit and water consumed were similar, those regarding average amounts of SSB consumed were dissimilar. These discrepancies could be due to children being more aware of when and how much fruit and water they consume than how much SSB they consume [8, 9, 11]. Fruit and water may be more straightforward than SSB. Although the definition of SSB was explained to the children and examples of SSB were provided, the children may still not have completely understood what SSB is. After all, it is more difficult to recall your consumption behaviour if you do not fully understand what it is you were supposed to remember, for example when remembering which drinks to include when answering certain questions. As a further explanation for such misreporting, one could consider the argument that healthier food or drink types such as fruit and water are more likely to be over-reported than unhealthy food or drink types such as SSB. This has been addressed by Börnhorst and colleagues in the context of intentional selective misreporting by parents (of 6101 children aged 2–9; mean age 6.1 (SD 1.8)): this study found that foods perceived as unhealthy (such as sugary products) were more likely to be under-reported whereas foods perceived to be more healthy (such as fruit and vegetables) were less likely to be under-reported [3]. So it would seem that there is a tendency towards over-reporting of healthier habits and towards socially desirable answers by parents [36, 37]. This might explain why, in the current study, average amounts of SSB consumed was dissimilar between parent and child reports while average amounts of fruit and water consumed was not. Yet another possible explanation for the discrepancy between child and parent report could be that children could have bought or swapped food items, without their parents knowing. In particular, this could especially be the case for items such as SSB’s. However, the potential over-reporting of healthy foods by parents could mean in our case that water and fruit intake reports of parents are higher than the true intake. Yet, children and parents reports on the child’s intake of fruit and water were similar. The role of social desirability on the answers of parents or children therefore stays obscure. Therefore, further research is required to address whether there are indeed differences between parents’ and children’s reports of healthy and unhealthy consumption in children.

The time between the child and parent reports ranged from one day to two months. Theoretically it is possible that seasonal influences on eating patterns or changes in feeding behaviours could have contributed to the discrepancies between self-reports of parents and children. Because our questions were on daily consumption and average amounts consumed, we assume that if this time delay between reports of parents and children was of influence, it will be of small influence since habit strength in consumption and feeding patterns tends to be high [38].

Others have looked for explanations for the discrepancies seen between parents and children. For example, McPherson and colleagues [8] suggest that one of the reasons that parents and children report different intakes is that they have different perceptions of the child’s food and drink intake. Additionally or alternatively, the discrepancy between parents and children could be explained by reporting bias, with parents reporting socially desirable answers more frequently than children [3].

A possible explanation for the higher reports of snacks as break-time food item (and possible over-reporting of SSB) by children could be due to personal preferences or personal characteristics, for example. Since snacks (and SSB) are more likely to be children’s favourite food (or drink) types, this could have been reflected in their reporting behaviour. In addition, we found statistically significant differences between the children’s reports of obesity-related foods and beverages and that observed or reported by parents, however hereby we contradict the findings found by Bennett and colleagues [36].

Previous research among parents and children reporting on child’s consumption behaviour has found that parental reports are slightly more accurate than children’s reports: 78 % compared with 72 % agreement regarding different food items and food types (children under study between the ages of 6 ang 11 years old) [15]. Given the differences and low levels of agreement that we found, we would recommend combined measurement methods when assessing child’s habitual dietary behaviour on the level of pre-specified foods or food groups. This is also suggested by Eck and colleagues who concluded in their study that the children’s contribution could be of value, given that the combined parent-and-child report was more accurate that the individual parent report (children under study between the ages of 4 and 10 years old) [7]. Reports from multiple sources are therefore recommended over single-source reports, especially for children younger than the age of 8 years old as proposed by Burrows and colleagues [4]. As seen in other public health fields, the question then remains on how to process data from multiple sources [39].

### Strengths and limitations

One of the strengths of this study is that it provides a unique assessment of different types of behaviour consumption in an ethnically diverse population. A further strength is the combined use of three measures – observations, parent questionnaires and child questionnaires – which provides insight into the added value of the various different assessment methods.

However, there are also limitations which should be acknowledged. Firstly, a maximum period of up to two months could have elapsed between completion of the child questionnaire and the parent questionnaire. Although our questions considered daily consumption and average amounts consumed, the time delay between parent and child reports has to be acknowledged when interpreting the results. Also, the data collection took place in the months April and May. As mentioned before, a seasonal effect cannot be ruled out, but given the timing of the measurements, we assume this is unlikely. Secondly, the questionnaire was provided in Dutch only, which could have been a barrier for some parents given the diverse ethnicity of our study population. Although we provided parents and children with definitions and examples, there may have been some confusion as to what constitutes the different snack and SSB categories. For instance, some members of the general population may not be aware of the differences between fruit juices such as apple juice and sweetened dairy products or energy drinks. Also, mis-categorisation by observers and children could have occurred when reporting on the brought food items. Lastly, regarding the parent-child comparison, we do not have an objective measurement of the reported intake and therefore cannot say anything about the ‘true’ intake, which is a limitation of the utility of that data.

## Conclusion

Children not only report higher than observed on amount of break-time foods of sandwiches and snacks, they also report a higher SSB intake than that reported by their parents. However, children and parents have similar estimations of the child’s water and fruit consumption. Since the level of agreement between the observed break-time foods and that reported by children and the agreement of child’s intake between parent and child reports are relatively weak, future studies should focus on improving methods of evaluating children’s consumption behaviour or on ways on how to best use and interpret multiple-source dietary intake data**.**

## Additional files

Additional file 1:
**S1.** Observation form. **S2.** Questionnaire item child. **S3.** Table: Overview of items used to assess child’s SSB intake in the parent and child questionnaire. **S4.** Bland Altman plots. 3a. Bland Altman plot – Water (L). 3b. Bland Altman plot – Fruit (# pieces). 3c. Bland Altman plot – Sugar-sweetened beverages (L) (PDF 731 kb) Additional file 2: STROBE checklist. (DOCX 29 kb)

## Abbreviations

ICC: intra-class correlation coefficients
SSB: sugar-sweetened beverages
