# Supplementary material for: Do children report differently from their parents and from observed data? Cross-sectional data on fruit, water, sugar-sweetened beverages and break-time foods
Source: BMC Public Health. 2016 Apr 18;16:341. doi: 10.1186/s12889-016-2963-7 (PMC4836148; doi:10.1186/s12889-016-2963-7)
Supplement: Additional file 1: — S1. Observation form. S2. Questionnaire item child. S3. Table: Overview of items used to assess child’s SSB intake in the parent and child questionnaire. S4. Bland Altman plots. 3a. Bland Altman plot – Water (L). 3b. Bland Altman plot – Fruit (# pieces). 3c. Bland Altman plot – Sugar-sweetened beverages (L) (PDF 731 kb) [file 12889_2016_2963_MOESM1_ESM.pdf]

Supplement 1. Observation form

School:	Grade:			Date:			Observer:			
**Student**	1	2	3	4	5	6	7	Etc.	…	…
**Food items**										
Nothing										
Fruit										
Vegetables										
Sandwiches										
Croissant, chocolate roll										
Rolls, bun, meusli bread										
Gingerbread, cracker										
Liga, Sultana										
Biscuits, cake										
Pie, muffin, pastry										
Candy, candy/chocolate-bars										
Chips, cheese, nuts										
Other, namely…										

Supplement 2. Questionnaire item child

Break-time Foods

What kind of foods did you brought to school to day to consume during break-time?

(You may pick ***more*** *than one answer!)*

☐ Nothing
☐ Fruit
☐ Vegetables
☐ Sandwiches
☐ Croissant, chocolate roll
☐ Rolls, bun, meusli bread
☐ Gingerbread, cracker
☐ Liga, Sultana
☐ Biscuits, cake
☐ Pie, muffin, pastry
☐ Candy, candy/chocolate-bars
☐ Chips, cheese, nuts
☐ Something else, namely: …

Supplement 3. Bland Altman plots

3a. Bland Altman plot – Water (L)

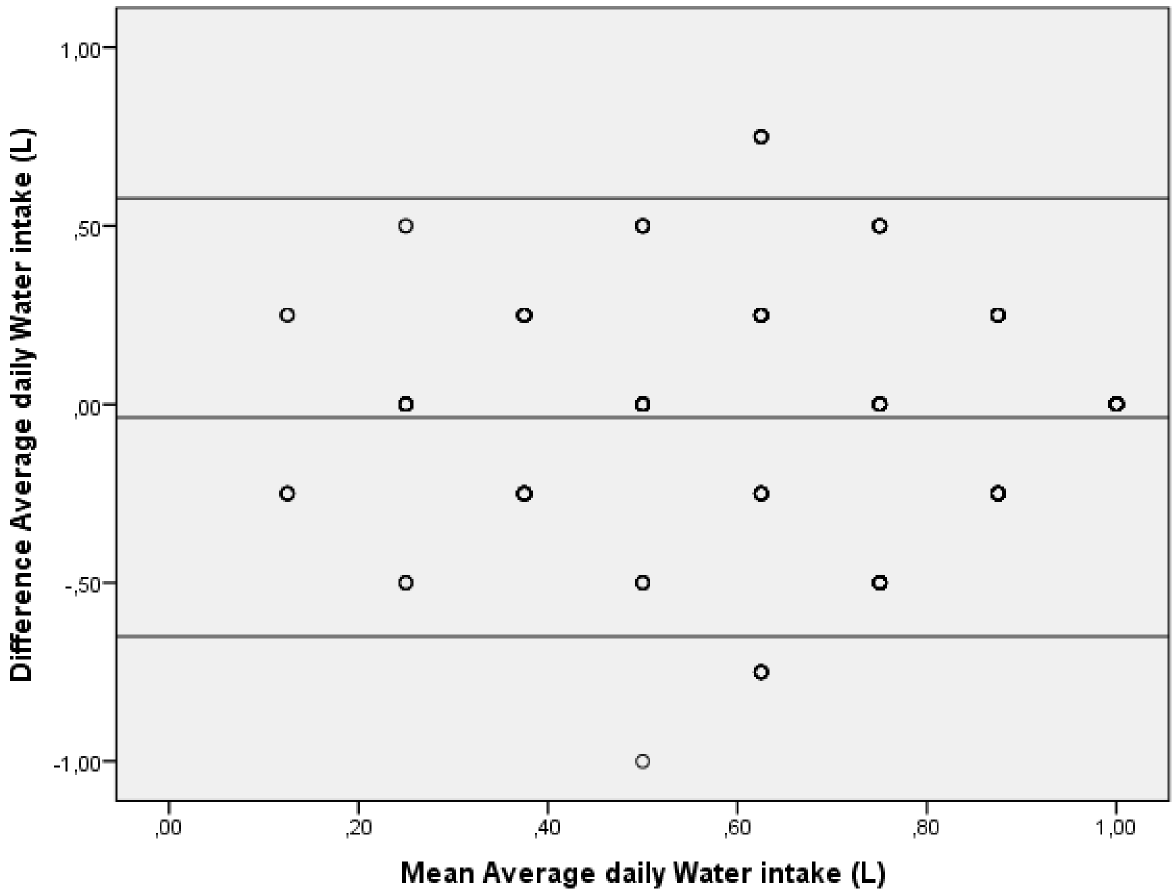

3b. Bland Altman plot – Fruit (# pieces)

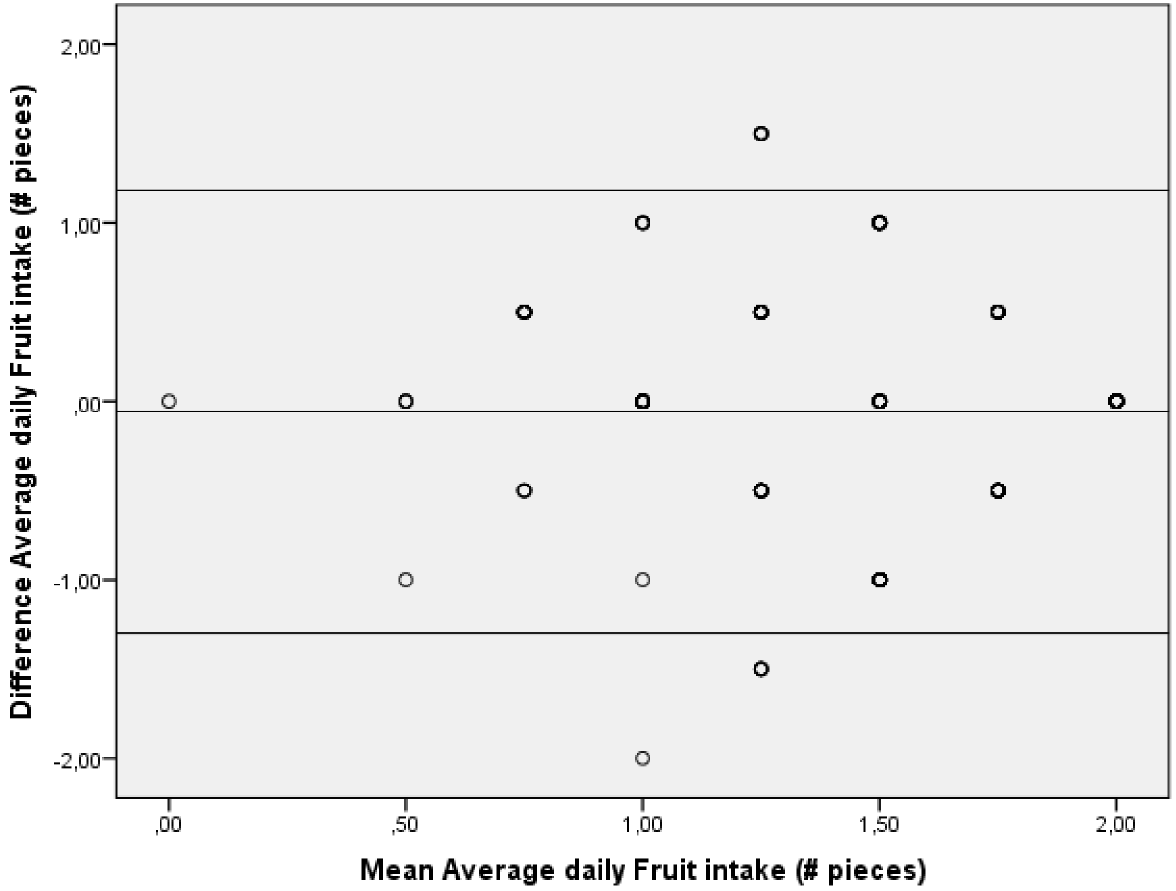

3c. Bland Altman plot – Sugar-sweetened beverages (L)

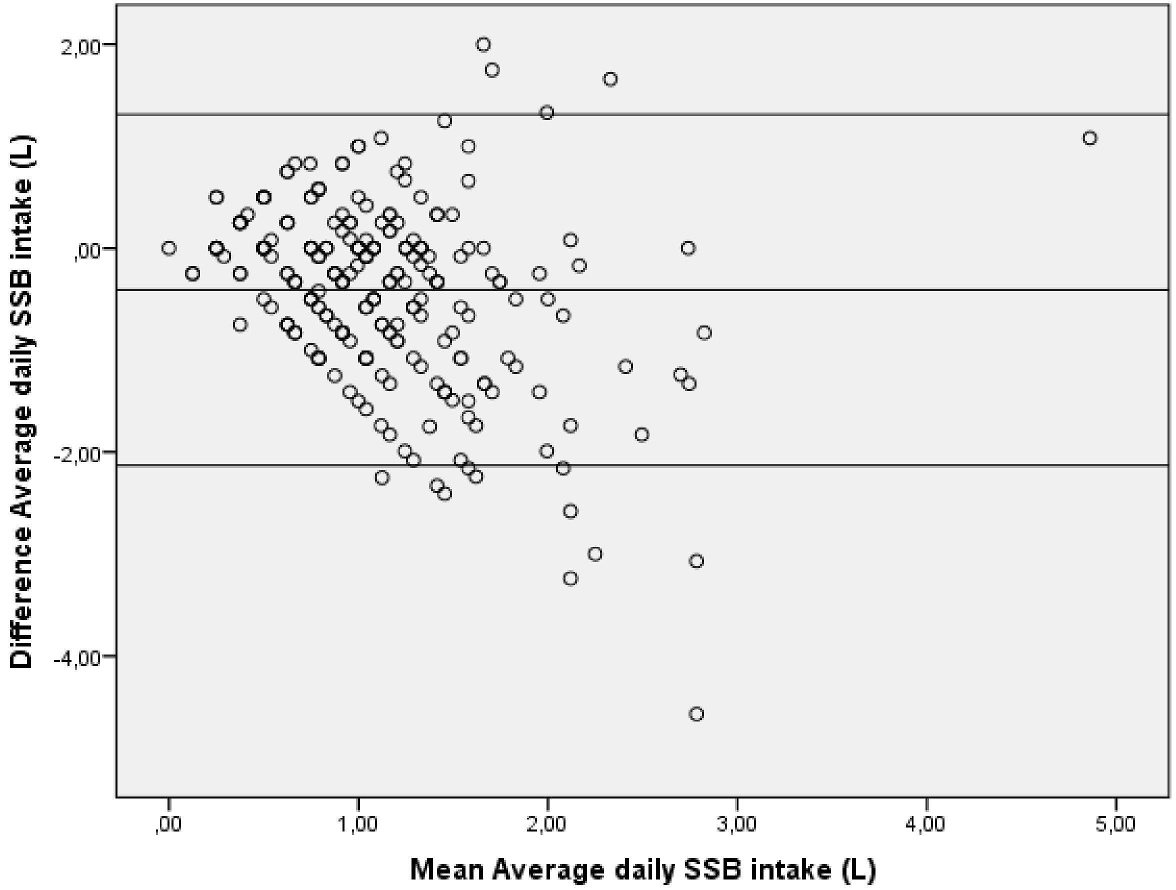

Supplement 4. Table: Overview of items used to assess child's SSB intake in the parent and child questionnaire

**Questionnaire items to assess child's SSB intake**	**Response categories**
Introduction to questions related child's SSB intake	
• Please indicate which of the drinks below your child (you) consume most of the times; this can be at school, at home or with friends.	☐ Coke/Pepsi<br>☐ Fanta/Sisi<br>☐ Fernandez<br>☐ Dr. Pepper<br>☐ Ice-tea<br>☐ Energy drinks (Redbull etc)<br>☐ Lemonade<br>☐ Apple juice<br>☐ Yoghurt-drinks<br>☐ Chocolate milk<br>☐ Tea with sugar
All questions below are related to so-called sugar-sweetened beverages (SSB). These are beverages containing added sugar, sweetened dairy products (e.g. chocolate milk), fruit juice (e.g. apple juice), soft drinks (e.g. cola) and energy drinks (e.g. sport energy drinks).<br><br>*All of the above examples of drinks are SSB.*<br>*Please fill in the questions below on how much SSB your child/you consume and keep above described definition and examples in mind.*<br><br>*(So, do not take into account: light or sugar free beverages, water, 100% orange juice, tea without sugar, and regular milk.)*	
• Does your child (do you) consume SSB on a daily basis?	☐ No, never<br>☐ No, not every day<br>☐ Yes, every day
• Please indicate how many glasses (250ml – column A), cans (330ml – column B) or bottles (500ml – column C) the child (you) consumed on an average day on which the child drank SSB?	**A. Glasses** 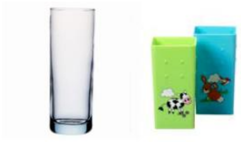<br>☐ None<br>☐ 1 glass<br>☐ 2 glasses<br>☐ 3 glasses<br>☐ 4 glasses<br>☐ 5 glasses<br><br>**B. Cans** 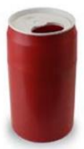<br>☐ None<br>☐ 1 can<br>☐ 2 cans<br>☐ 3 cans<br>☐ 4 cans<br>☐ 5 cans<br><br>**C. Bottles** 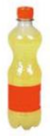<br>☐ None<br>☐ 1 bottle<br>☐ 2 bottles<br>☐ 3 bottles<br>☐ 4 bottles<br>☐ 5 bottles
